# Single-cell analysis of myeloid cells in HPV^+^ tonsillar cancer

**DOI:** 10.3389/fimmu.2022.1087843

**Published:** 2023-01-19

**Authors:** David Gomez Jimenez, Can Altunbulakli, Sabine Swoboda, Aastha Sobti, David Askmyr, Ashfaq Ali, Lennart Greiff, Malin Lindstedt

**Affiliations:** ^1^ Department of Immunotechnology, Lund University, Lund, Sweden; ^2^ Department of ORL, Head & Neck Surgery, Skåne University Hospital, Lund, Sweden; ^3^ Department of Clinical Sciences, Lund University, Lund, Sweden; ^4^ National Bioinformatics Infrastructure Sweden, Science for Life Laboratory, Lund University, Lund, Sweden

**Keywords:** tonsillar cancer, human papilloma virus, myeloid cell, dendritic cell, single-cell RNA-sequencing, macrophage

## Abstract

The incidence of human papillomavirus-positive (HPV^+^) tonsillar cancer has been sharply rising during the last decades. Myeloid cells represent an appropriate therapeutic target due to their proximity to virus-infected tumor cells, and their ability to orchestrate antigen-specific immunity, within the tonsil. However, the interrelationship of steady-state and inflammatory myeloid cell subsets, and their impact on patient survival remains unexplored. Here, we used single-cell RNA-sequencing to map the myeloid compartment in HPV^+^ tonsillar cancer. We observed an expansion of the myeloid compartment in HPV^+^ tonsillar cancer, accompanied by interferon-induced cellular responses both in dendritic cells (DCs) and monocyte-macrophages. Our analysis unveiled the existence of four DC lineages, two macrophage polarization processes, and their sequential maturation profiles. Within the DC lineages, we described a balance shift in the frequency of progenitor and mature cDC favoring the cDC1 lineage in detriment of cDC2s. Furthermore, we observed that all DC lineages apart from DC5s matured into a common activated DC transcriptional program involving upregulation of interferon-inducible genes. In turn, the monocyte-macrophage lineage was subjected to early monocyte polarization events, which give rise to either interferon-activated or CXCL-producing macrophages, the latter enriched in advanced tumor stages. We validated the existence of most of the single-cell RNA-seq clusters using 26-plex flow cytometry, and described a positive impact of cDC1 and interferon-activated DCs and macrophages on patient survival using gene signature scoring. The current study contributes to the understanding of myeloid ontogeny and dynamics in HPV-driven tonsillar cancer, and highlights myeloid biomarkers that can be used to assess patient prognosis.

## 1 Introduction

Tonsillar squamous cell carcinoma, or short tonsillar cancer (TC), is a head and neck cancer (HNC) caused by abnormal growth of epithelial cells of the tonsillar mucosa. Risk factors include tobacco smoking and alcohol abuse as well as high-risk human papillomavirus (HPV) infection (1). The incidence of HPV^+^ TC is sharply rising (2) and currently accounts for > 70% of TC cases (3, 4). Biomarkers associated with the immune compartment of the tumor microenvironment (TME) have shown prognostic value in HPV^+^ TC. For instance, several studies have highlighted a correlation between tumor-infiltrating CD8^+^ T-cells, T regulatory cells (Tregs) (5, 6), and PD-L1 expression (7, 8), respectively, to clinical outcome. However, immune-checkpoint blockade strategies, aiming at inducing T-cell responses, have shown limited clinical efficacy in HNCs, irrespective of HPV status (9, 10). A different approach to treat cancer concerns harnessing anti-tumor immunity by targeting myeloid cells due to their ability to modulate lymphoid cell activity (11). Increased knowledge about myeloid cell heterogeneity, their subset-specific features and functions within the TME of HPV^+^ TC, and their potential impact on survival is warranted in order to develop new treatment strategies.

Myeloid cells are divided into conventional dendritic cells (cDCs), monocytes, macrophages, and granulocytes, each characterized by cell-type specific functions (12, 13). DCs act as sentinel cells in peripheral tissue by taking up antigens, transferring them into lymphoid organs, and triggering T-cell activation (12). In the context of HNC, both cDC1 and cDC2 express CCR7 at protein level, highlighting their migratory potential (14). Furthermore, cDC2 subsets were specifically shown to induce and correlate with Th1 CD4^+^ T-cell abundance in the TME (14). In contrast, cDC1 migrate *via* XCL1 and CCL5, presumably produced by tumor infiltrating NK-cells (15). Using a combination of gene-signature scoring in bulk HNC RNA-seq, these chemokines were shown to correlate with both cDC1 and NK-cell signatures (15), stressing the relevance of the cDC1-NK-cell axis in HNC. Human cDC1s are known for their superior capacity to mount anti-tumoral CD8^+^ T-cell responses through antigen cross-presentation (16). However, *in vitro* studies have shown that human cDC2 from blood and tonsils also can cross-present antigens upon appropriate stimulation (16, 17). Compared to DCs, macrophages are sensors of tissue damage and infection, which help in clearing apoptotic cells and activating T-cells *in situ*. The functional role of macrophages in HNC is not clarified, and several retrospective IHC studies report contradictory results regarding prognostic impact (18–21). Studies of other cancers have presented macrophages as highly plastic cells involved in extracellular matrix remodeling through MMPs (22), angiogenesis *via* VEGF-α production (23), and attenuation of immune responses through TGF-β (24). Finally, monocytes can have both pro- and anti-tumoral functions, and differentiate into monocyte-derived DCs (25, 26) and tumor-associated macrophages (11, 27–29).

Recently, myeloid cell diversity has been revisited through the use of single-cell RNA-sequencing (scRNA-seq) in blood (30, 31), tonsil (32), and spleen (33) as well as in a variety of tumors (33–35), tumor draining lymph nodes (14, 36), and peritoneal ascites from cancer patients (26, 28). Although these studies report highly overlapping DC subsets, they also highlight differences in DC biology depending on the tissue of origin. In blood, the DC compartment, in addition to cDC1 and cDC2, also consists of the CD14^+^ CD163^+^ DC3 and Axl^+^ Siglec6^+^ DC5 subtypes (30). Analyses of the DC lineage in inflamed tonsil and spleen have demonstrated three additional populations closely related to cDC2s: a small proliferating DC cluster, a CLEC10A^-^CLEC4A^+^ cDC2 population, and an activated CCR7^+^ cDC2 subset (26, 33). The heterogeneity of the monocyte-macrophage lineage (Mono-Macs) is even greater, including up to 15 different communities in a recent cross-tissue meta-analysis (35). The diversity of the Mono-Mac lineage is partly related to the disparity of markers and clusters reported, but also due to the low resemblance of *in vivo* Mono-Macs with the well characterized *in vitro* M1/M2 macrophage models (37). Additionally, macrophage ontogeny has been postulated to condition Mono-Mac function, and tissue resident macrophage populations such as Langerhans cells (LC) have recently gained attention (36, 38, 39). Collectively, the divergences observed in scRNA-seq studies possibly relate to the differentiation and maturation of myeloid cells, which in tissue are greatly influenced by the local microenvironment.

Myeloid cells represent potential therapeutic targets in TC since they can orchestrate anti- and pro-tumor T-cell responses. Given their divergent T-cell polarization capacity (26, 31, 32, 40), there is a need to profile their diversity within the TME and at healthy steady state settings. Several groups have assessed the transcriptional profiles of the whole immune compartment of HNC biopsies using scRNA-seq (41–43). However, these studies are limited in their capacity to resolve myeloid cell heterogeneity due to low frequency of these cells in the TME. Furthermore, HNCs arise in different anatomical sites and are heterogeneous in terms of immune-infiltration, and thus, it is important to delineate tumor subsite-specific nuances. Lastly, published studies most often use blood or tonsil specimens from patients with local inflammation as control material, which are informative, but may not represent appropriate steady state lymphoid tissue controls.

In this study, we evaluate myeloid cell heterogeneity and maturation status in HPV^+^ TC (*cf*. paired healthy tonsils (HT)), and the impact of these features on survival of HNC patients. We implement droplet-based 10X Genomics scRNA-seq to characterize FACS-sorted CD45^+^CD13^+^HLA-DR^+^ myeloid cells, describing transcriptomically unique clusters, which include novel cDC2, LC, and Mono-Mac populations. Notably, we describe a preferential recruitment of pre-cDC1s in the TME, giving rise to cDC1s that mature into activated DCs. We further show that patients enriched in gene-signatures from cDC1s and interferon-activated DCs and macrophages have a higher five-year overall survival. Better understanding of the myeloid complexity and functionality in TC can improve the design of myeloid-targeted therapies, which potentially can overcome the limitations of T-cell oriented immunotherapies in TC.

## 2 Materials and methods

Collection of tumor and contralateral tonsillar tissue was approved by the Swedish Ethical Review Authority (ref. no. 2017/580), and all participating patients granted written informed consent. Paired biopsies from TC (n=15) and contralateral HT (n=8) were obtained at Lund University Hospital at the time of diagnosis (**Table S1**).

### 2.1 Cell isolation

TC and HT samples were cut into small fragments in RPMI 1640 medium (Thermo Fisher Scientific, Bremen, Germany) supplemented with 0.1 mg/mL gentamycin (Sigma-Aldrich, St Louis, MO). The tissue fragments were enzymatically digested with Collagenase IV (Sigma-Aldrich) (2.0 mg/mL) and DNase I (Sigma Aldrich) (200 Kunits/mL) for 20 minutes at 37°C. Cells were filtered using a 70 μm cell strainer (BD Biosciences, San Jose, CA). For scRNA-seq, CD45^+^ CD13^+^ HLA-DR^+^ myeloid cells were isolated using FACSAria IIu (BD Biosciences) by cell sorting into FACS tubes containing 3 mL of fetal calf serum (FCS) (Life Technologies, Carlsbad, CA). Sorted myeloid cells were washed twice and resuspended in 45 μL of PBS supplemented with 0.04% w/v bovine serum albumin (BSA).

### 2.2 Flow cytometry

Cells were stained with Fixable viability stain 620 (BD Biosciences) to assess cell viability. Cells were washed and non-specific binding was blocked with ChromPure mouse IgG whole molecule (Jackson ImmunoResearch, West Grove, PA) for 15 minutes at room temperature. Cells were immediately stained with the appropriate antibody panel (**Tables S2**, **S3**) for 20 min at 4°C, washed, and analyzed on a FACSAria IIu instrument (BD Biosciences) or Cytek Aurora (Cytek Biosciences, Fremont, CA).

### 2.3 scRNA-seq library preparation and sequencing

The scRNA-seq libraries were prepared according to the user guide manual (CG000204) provided by 10X Genomics’ Chromium Single Cell 3’Reagent Kit (v3.1) (10X Genomics, Pleasanton, CA). Briefly, sorted cellular suspensions of myeloid cells were loaded on a Single Cell chip to encapsulate single cells using the 10X Chromium controller. Encapsulated cells were then subjected to in-drop lysis and reverse transcription reactions. The emulsion droplets were disrupted, and barcoded cDNA was purified using silene magnetic dynabeads (Thermo Fisher Scientific, Waltham, MA), followed by 12-14 cycles of PCR amplification using a C1000 Touch™ Thermal Cycler with 96–Deep Well Reaction Module (#1851197, Bio-Rad. Hercules, CA). The amplified cDNA was purified with the SPRIselect reagent kit (Beckman Coulter, Brea, CA) and subjected to enzymatic fragmentation, end repair, A-tailing, size selection with SPRIselect, adaptor ligation, post-ligation cleanup with SPRIselect, and sample index PCR followed by cleanup with SPRIselect. Library size and quality were assessed with High Sensitivity D1000 ScreenTape using a 4200 TapeStation System (G2991BA, Agilent, Santa Clara, CA). The cDNA libraries were sequenced on NovaSeq 6000 System (R1 – 28, i7 8, R2 – 91 cycles, Illumina, San Diego, CA).

### 2.4 scRNA-seq data analysis

Pre-processing of scRNA-seq was performed using Cell Ranger (v6.0). This pipeline included sample de-multiplexing, barcode processing, and 3’ gene counting. Reads were aligned to the GRCh38 transcriptome (v. 2020-A) and single cells within each sample were merged using the aggregate function in Cell Ranger. A total of 11,663 single cells with an average of 92,500 raw reads per cell were further analyzed in R (v4.0.3) and R studio (v1.4.1103) using the Seurat package (v4.0.1) (44). The datasets are available at GEO (GSE219210). A full collection of the pipeline that replicates the data analysis outlined in this manuscript is available as an R script repository on Github (https://github.com/dgomjim/Code_scRNAseq_myeloid_cells_in_HPV_TC).

We used Seurat to perform thresholding, normalization, integration, dimensionality reduction, cluster identification, and visualization as well as differential gene expression analysis. The gene-barcode matrix was filtered selecting cells with more than 700 genes and less than 6,000 genes detected per cell. Cells with more than 10% mitochondrial and more than 50% ribosomal UMI-counts were filtered out. Successful removal of doublets was ensured by comparing filtered cells to the barcodes selected using the DoubletFinder package (v2.0.3). Contaminating cells were identified and filtered out based on their expression of T-cell, B-cell, and NK-cell canonical genes (e.g., *CD3D* < 0.1 & *CD3E* < 0.1 & *CD8A* < 0.1 & *CD8B* < 0.1 & *TIGIT* < 0.1 & *CD19* < 0.1 & *MS4A1* < 0.1 & *KLRB1* < 0.1 & *CD7* < 0.1 & *GNLY* < 0.1 & *CTLA4* < 0.1). Additionally, mitochondrial, and ribosomal genes were excluded before proceeding to data merging and normalization by log-transformation. Cell cycle scores were calculated using the “CellCycleScoring” function within Seurat, which generates a module score based on pre-set gene-sets characteristic of S, G2, and M phases of the cell cycle. Datasets from different samples were merged, and sample specific IDs were added to cellular barcodes allowing sample-based discrimination of cells during downstream steps. A total of 9,505 single cells were further selected for sample integration, which was performed *via* canonical correlation analysis (CCA) using the top 2,000 highly variable features across the datasets. The gene-barcode matrix was then scaled, and regression of gene expression was conducted based on UMI-counts and total number of unique UMIs as well as the percentage of mitochondrial and ribosomal genes. Principal component Analysis (PCA) was then performed on the normalized and scaled gene-barcode matrix. The first 20 principal components were selected for non-linear dimensionality reduction (tSNE and UMAP) based on their variance using the Elbow Plot approach. The shared nearest neighbors (SNN) algorithm was used to calculate distances between cells. Cell clusters were calculated using the community detection algorithm smart local moving (SLM). We inspected the granularity of the clusters, using step-wise increments of 0.1 in the resolution parameter (0.1-1) implemented in the “FindClusters” function, and plotted them using the ClustTree package (v0.4.3) (45). Differential gene expression analysis across cell clusters was performed using the “FindAllMarkers” function in Seurat. We applied Wilcoxon rank sum test to perform the analysis, only accounting for genes overexpressed in at least 10% of the cells in a cluster (p-value < 0.01, log_2_FC > 1).

### 2.5 Gene signature analysis

We computed signature scores using the “AddModuleScore” function in Seurat to assess cluster identity. Scores were calculated by binning signature genes in 25 bins according to average expression. The signature’s average expression was corrected by subtracting the aggregated expression of 100 randomly selected genes from the same bin. We used published gene signatures for blood cDC1, cDC2, DC3, DC4, DC5, DC6, and monocytes 1-4 (30), *in vitro* activated cDC2 and moDC (26), M1/M2 *in vitro* polarized macrophages (37), skin LC (39), monocytes and monocyte-derived DC and macrophages (11, 35), myeloid derived suppressor cells (MDSC) (46), and tissue mast cells (35). Additionally, enrichment scores from a pre-annotated bone marrow single cell dataset (47) were computed across our myeloid clusters in order to identify progenitor DCs. The CITE-seq bone marrow dataset was accessed through the human cell atlas (48), and a supervised PCA was computed. Supervised PCA was used to map our query myeloid dataset onto the multimodal bone marrow reference, obtaining enrichment scores for each annotation.

### 2.6 Single-cell regulatory network inference and clustering analysis

We used single-cell regulatory network inference and clustering (SCENIC) to detect and score regulons in single cells (49). The pipeline consisted of three steps: generation of a co-expression modules using Genie3, module filtering based on enrichment of DNA binding sites using RcisTarget databases, and estimation of regulon activity with AUCell. The relative activity of the top ten enriched regulons per cluster was used to generate a heatmap. The regulon heatmap was complemented with a dot plot displaying the relative gene expression of the corresponding TF.

### 2.7 Developmental pathway analysis

We used the Velocyto python package to recount the spliced and unspliced reads. Selection of highly variable genes was performed on total reads, followed by a filtering step to select genes with more than 20 counts on both spliced and unspliced assays. After filtering, top 400-500 highly variable genes were used to calculate RNA velocity applying the scVelo “stochastic” model in the R package Velociraptor (50). Finally, we embedded the velocity vector in either a diffusion map (51) or UMAP to visualize developmental trajectories. The frequency of maturing DCs was calculated as the percentage of DCs with high RNA velocity values transitioning into LAMP3+ expressing actDC, out of the total number of DC in each lineage.

### 2.8 Survival analysis

TCGA-HNSC normalized gene expression data as well as clinical metadata were downloaded from the GDC portal repository of the cancer genome atlas (TCGA) (52). Individual samples were normalized and z-scored for cluster-specific gene-sets using the GSVA package (53). Then, samples were divided into quartiles, and we defined high (Q1) and low (Q4) enriched samples for each cluster-specific gene set. We performed five-year overall survival analysis using the survival and survminer packages (54). Difference between survival curves were assessed by log-rank test (p-value < 0.05). We used uni- and multivariate Cox proportional-hazards models to estimate the relation between the gene-signature scores and the mortality rate. The assumptions of the Cox proportional-hazards model were tested assessing the Schoenfeld residuals.

### 2.9 Pathway analysis

Gene set enrichment analysis (GSEA) was performed using EnrichR (v3.0) (55) by querying differentially expressed genes among myeloid clusters onto the gene ontology database (GO, GO_bioprocess_2018) (56). We selected reoccurring and highly ranked pathways (adj.p-value < 0.01) related to APC function. We harmonized these pathways throughout clusters using gene set variation transformation (53). The resulting GSVA scores were plotted as a clustered heatmap using pheatmap (v1.0.12).

### 2.10 RL interaction analysis

To elucidate potential RL interactions of myeloid cells, we built a “meta-dataset” containing our in-house dataset and tumor infiltrating leukocytes from TC and HT samples of a publicly available HNC scRNA-seq dataset (41). In brief, we subsetted single cells from three TCs and five HT samples and performed normalization, sample integration *via* CCA, data scaling, dimensionality reduction, cluster detection, and annotation based on canonical markers. Next, we integrated leukocytes of the external and in-house datasets, normalized them *via* “SCTransform”, and re-calculated PCA and UMAP. Once we obtained the harmonized “meta-dataset”, we proceeded to RL analysis using the R package CellChat (57). First, we identified RL interactions enriched in specific clusters in TC and HT separately. To do so we split the “meta-dataset” into cancer and healthy, and selected overexpressed genes detected in at least 25 of the cells per cluster (p-value < 0.05, FC > 2) before calculating the communication probability. Next, we merged the cancer and healthy CellChat objects and filtered cluster specific RL interactions that were enriched in cancer. Finally, we visualized cluster specific RL interactions of cDC1s, actDCs, and act Macros with subsets of T/NK-cells using the “netVisual_bubble” function in the CellChat package.

## 3 Results

### 3.1 Expansion of the myeloid compartment in HPV^+^ TC include subsets of DCs, monocytes, macrophages, and a small population of HLA-DR^+^ granulocytes

**Figure 1:**
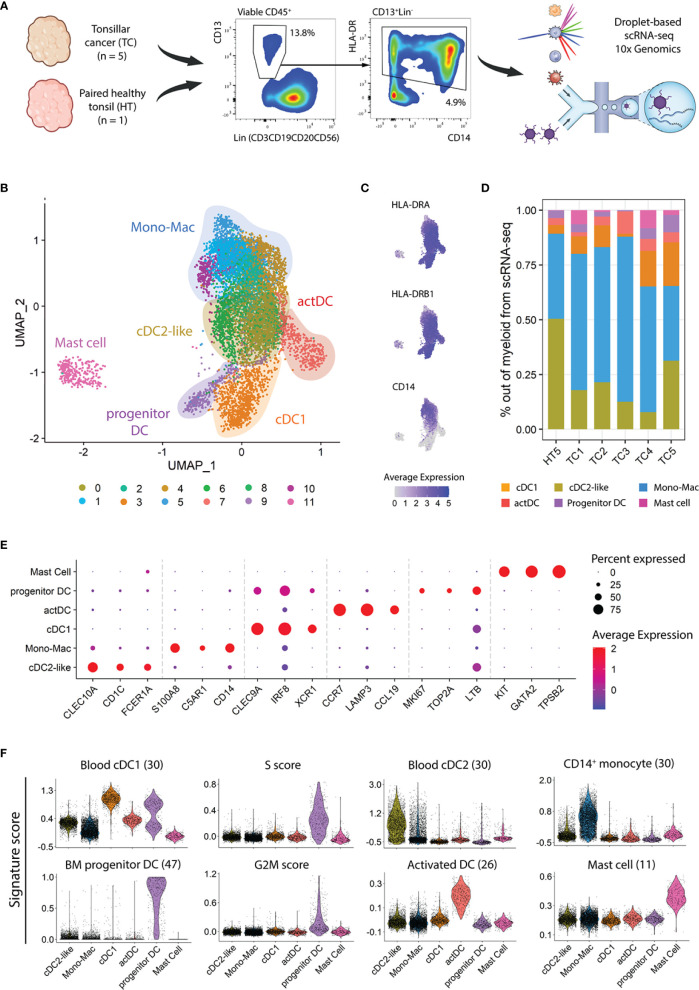
Heterogeneity of myeloid populations in TC and HT assessed by scRNAseq. **(A)** Viable CD45^+^ CD13^+^ HLA-DR^+^ myeloid cells were sorted from single cell suspensions of five TC samples and one contralateral HT and subjected to scRNA-seq using 10X genomics. The frequencies in the dot plots represent the average in TC calculated from viable CD45^+^ cells. **(B)** UMAP visualization of single cell transcriptomes displaying twelve clusters grouped into six major myeloid subtypes. **(C)** UMAP visualization, as in B, showing expression levels of *HLA-DRA*, *HLA-DRB1*, and *CD14*. **(D)** Frequency of myeloid populations across tissue of origin. **(E)** Dot plot displaying three canonical markers among the top ten differentially expressed genes across clusters. **(F)** Violin plots showing scores of indicated signatures. The score in each cell is shown in relative units, along with the density distribution shown by the shape of the plot. G2M and S scores represent the relative expression of cell cycle phase genes in a cell. Scale bars represent normalized counts in **(C)** and **(E)**.

To evaluate the heterogeneity of the myeloid compartment in HPV^+^ TC, we dissociated fresh biopsies into single cells and used flow cytometry to characterize and quantify CD45^+^CD13^+^HLA-DR^+^ myeloid cell populations (**Figure S1A**). We observed a significant increase in the frequency of CD13^+^HLA-DR^+^ cells in TC (n=8) as compared to contralateral HT (n=5), indicating that the myeloid compartment expands in TC (**Figure S1B**). Within the myeloid gate, the greatest frequency increase in TC as compared to HT was observed in the Mono-Mac and LC populations, followed by cDC1s. Next, we addressed the diversity of myeloid cells in treatment-naïve TC patients at transcriptomic level. We sorted CD45^+^CD13^+^HLA-DR^+^ cells from five HPV^+^ TC biopsies and one paired contralateral HT, followed by scRNA-seq using the droplet-based 10X Genomics platform (**Figure 1A**). After QC, normalization, and sample integration, we used UMAP to visualize single cells in a low dimensional space. Unsupervised clustering of 9,502 single cells revealed 12 clusters (**Figure 1B**; **Figure S1C**), characterized by expression of MHC class II coding transcripts (e.g., *HLA-DRA*, *HLA-DRB1*) and heterogeneous expression of *CD14* (**Figure 1C**), with different distribution in TC and HT (**Figure 1D**).

Next, we annotated the twelve clusters using a combination of canonical marker gene expression, after differential gene expression analysis (DGEA) across clusters and gene signature scoring (11, 26, 30, 47) (**Figures 1E, F**). The cDC1 population (cluster 3) expressed high levels of *CLEC9A, IRF8*, and *XCR1*, along with a high score of a described blood cDC1 gene-set. Similar to that observed at protein level (**Figure S1B**), the cDC1 frequency in the scRNA-seq dataset increased in TC as compared to paired HT. In turn, cDC2-like cells (clusters 0 and 6) displayed *CLEC10A*, *CD1C*, and *FCER1A* expression and enrichment in a blood cDC2 gene set. Two additional DC clusters were predicted, representing different cell states of DC development and maturation. Progenitor DCs (cluster 9) exhibited markers related to cell cycle and progenitor cells (*MKI67*, *TOP2A*, and *LTB*), as illustrated by their high S and G2/M cell cycle phase scores. Additionally, this cluster featured high prediction scores of bone-marrow DC progenitor cells as well as low scores of hematopoietic stem cells and granulocyte-monocyte progenitors (**Figure S1D**). The 4^th^ DC population, activated DC (actDC, cluster 7), was characterized by high expression of genes related to DC maturation, such as *CCR7*, *LAMP3*, and *CCL19*, and high scores for a gene-set from activated DCs. Six clusters of CD14^+^ cells (clusters 1, 2, 4, 5, 8, and 10) conformed to the Mono-Mac lineage (**Figures 1E, F**). The Mono-Mac population was characterized by expression of canonical genes *S100A8/9* and *C5AR1* as well as a high CD14^+^ monocyte signature score. As observed in the flow cytometry analysis (**Figure S1B**), Mono-Macs were more frequent in TC as compared to HT (**Figure 1D**). Lastly, cluster 11 showed high expression of *KIT*, *GATA2*, and *TPSB2* as well as an exclusive mast cell signature score, which only was detected in TC samples. Mast cells expressed lower levels of MHC class II related transcripts compared to other myeloid cells, and expression of co-stimulatory molecule coding genes was not detected (**Figure 1C**; **Figures S1E**).

### 3.2 DC lineage-specific subpopulations and analysis of cell identity dynamics.

**Figure 2:**
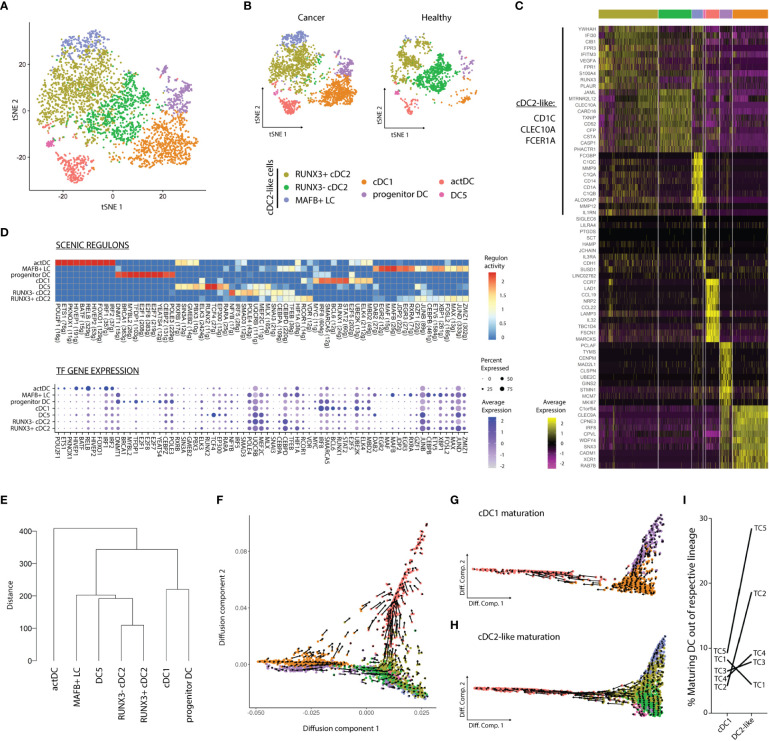
Dendritic cell dynamics and activation differ according to the tissue of origin. **(A)** tSNE plot showing seven distinct DC clusters DC clusters in TC and HT, or **(B)** separated by tissue of origin. **(C)** Heatmap displaying top ten DEG per cluster after DGEA (FC > 1.5, p-value < 0.05). **(D)** Heatmap of the top 10 regulons per cluster colored by regulon activity (upper) and dot plot displaying the gene expression levels of each regulon’s TF per cluster (lower). **(E)** Dendrogram of DC clusters based on the Euclidean distance. Diffusion map combined with RNA velocity analysis to infer the development trajectory of all DCs **(F)**, cDC1s **(G)**, and DC2-like cells **(H)**. **(I)** Frequency of maturating cDC1s and DC2-like cells annotated by sample of origin, as inferred from RNA velocity analysis. DEG, differentially expressed genes; DGEA, differential gene expression analysis; DC, Dendritic cell; TF, Transcription factor.

To detect rare populations, we divided the dataset into DCs and Mono-Macs and re-clustered these lineages separately. Re-clustering followed by DGEA revealed seven transcriptomically different communities in the DC lineages across TC and HT (**Figures 2A-C**; **Table S4**). Three clusters of cDC2-like cells were characterized by expression of *CLEC10A*, *CD1C*, and *FCER1A* (**Figure 2C**), high scoring of blood and tissue cDC2 gene-sets (**Figure S2A**), and heterogeneous distribution in TC and HT (**Figure 2B**). We identified two clusters of cDC2s with different expression of the transcriptional factor *RUNX3*. RUNX3^-^ cDC2s exhibited preferential expression of *CLEC10A, JAML*, and *TXNIP* and were only present in HT. In contrast, RUNX3^+^ cDC2s were characterized by expression of interferon-inducible genes (*IFI30*, *IFITM3*), and were more abundant in TC as compared to HT. Additionally, MAFB^+^ LCs uniquely expressed *CD1A*, *TGFB*, *CD14*, and *C1QA/B/C* and were only found in TC. This population was enriched in a LC and moDC gene-sets, but not in a previously described blood CD14^+^ DC3 gene-signature (**Figure S2A**). MAFB^+^ LC displayed high G2M scores, highlighting their self-renewal capacity and further confirming their identity as LC (38) (**Figure S2C**). A rare population of DC5s expressing *SIGLEC6*, *LILRA4*, and *IL3RA* was found closely associated with the cDC2-like cell cluster. DC5s featured a high blood DC5 gene-set score (**Figure S2A**) and an increased frequency in HT as compared to TC. Lastly, cDC1s, actDCs, and progenitor DCs corresponded to the three remaining DC populations, equally observed in the previous clustering (**Figure 1B**). The correspondence between the two clustering steps showed the homogeneity of cDC1, actDC, and progenitor DC communities at the present sequencing depth.

We used SCENIC to assess different activity of gene regulatory networks in our clusters (**Figure 2D**; **Table S5**). We noted that cDC1s progenitor DCs and actDCs featured the most distinct regulons in the DC compartment, while cDC2-like cells and DC5s partly shared their regulatory networks. actDCs were characterized by high activity of RELB, HIVEP1, and IRF1/2 regulons. In turn, cDC1s featured high activity, and expression of STAT2, RUNX1, and IRF8 regulons. Progenitor DCs uniquely displayed activity and expression of E2F1/7/8 as well as BRCA1 and MYBL2. DC5s uniquely showed TCF4 and RUNX2 regulon activity and expression, and preferential RARA enrichment as compared to MAFB^+^ LC. On the other hand, MAFB^+^ LC exhibited exclusive MAFB activity and expression, and preferential enrichment in RXRA and DAB2 among other regulons and TFs. Finally, RUNX3^-^, RUNX3^+^ cDC2s, and MAFB^+^ LC shared several active regulons including SNAI3, CEBPD, and TFEB. However, while RUNX3^+^ cDC2s exhibited higher activity of VDR, RUNX3^-^ cDC2s were characterized by higher NFYB and SMAD3 activity. Next, we performed hierarchical clustering based on Euclidean distance between the clusters to assess their transcriptomic similarity (**Figure 2E**). We observed that actDCs were the most divergent group, clustering separately in the dendrogram. cDC2-like cells and DC5s clustered together and were distant from the cDC1 and progenitor DC pair, in line with the results from our gene regulatory network analysis.

Next, we sought to study cell identity dynamics of DC communities. Thus, we projected single cell transcriptomes along with RNA velocity information onto diffusion map embeddings to infer future states of cells (**Figures 2F–H**). Overall, we observed directionality in RNA velocity of both cDC1 and cDC2-like cell branches towards actDCs. cDC1s and cDC2-like maturation into actDCs was confirmed by the sequential loss of *CLEC9A* and *CLEC10A*, respectively. These events were followed by a sequential increase in *LAMP3* expression along the trajectory, as observed in a 3D UMAP embedding (movie S1). The maturation of the cDC1 lineage was investigated by subdividing cDC1s along with progenitors enriched in cDC1 gene-set and actDC. RNA velocity analysis displayed a continuum of states along the progenitors into the fully differentiated cDC1 cluster, progressing to actDCs (**Figure 2E**). We evaluated transcriptomic changes along the developmental trajectories by assessing the velocity of highly variable genes with cluster-specific splicing dynamics (**Figure S2B**). We observed that progenitor DCs had higher unspliced levels of *DNAJB1*, *FUCA1* and *DNMT1*, on their development towards cDC1s. In turn, *SGO2* and *CTSL* were downregulated on the progression of progenitor DCs towards cDC1s and actDCs. cDC1s matured into actDCs displaying an initial upregulation of *CD40*, followed by *CCR7*, *PRDM1*, *NFKB1*, *LAMP3, IDO1*, and *MIR155HG*. In parallel, we analyzed cDC2-like cells likewise and detected a strong directionality from RUNX3^-^ cDC2s and MAFB^+^ LC into RUNX3^+^ cDC2s, which later progressed into actDCs (**Figure 2H**). We observed that all DC2-like clusters presented active transcription of *CD1C*, *IRF4*, and *HIVEP1*, and the last two genes were markedly upregulated upon maturation into actDCs (**Figure S2C**). MAFB^+^ LC and RUNX3^+^ cDC2s exhibited upregulation of *RUNX3*, while only RUNX3^-^ cDC2s upregulated *ISG20* upon progression into RUNX3^+^ cDC2s. MAFB^+^ LCs specifically downregulated *CD1A* and *CD207* upon progression into RUNX3^+^ cDC2s. DC5s displayed active transcription of *TCF4*, while *LILRA4* was downregulated. In turn RUNX3^+^ cDC2s sequentially upregulated *CD274*, *LAMP3*, and *TCF4*, followed by *IRF1*, upon maturation into actDCs. Contrary to progenitor cDC1s, we did not detect a directional flow in DC5s nor in CLEC10A^+^ progenitor DCs. Finally, we used RNA velocity information to estimate the frequency of cDC1 and DC2-like cells maturing into actDC expressing *LAMP3* (**Figure 2I**). We observed that DC2-like cells matured more frequently than cDC1s. We also noted that cDC1 and DC2-like maturation in the same TC samples did not correlate to each other, indicating that these two processes are independent.

### 3.3 Mono-Mac subsets display tumor-stage specific patterns

**Figure 3:**
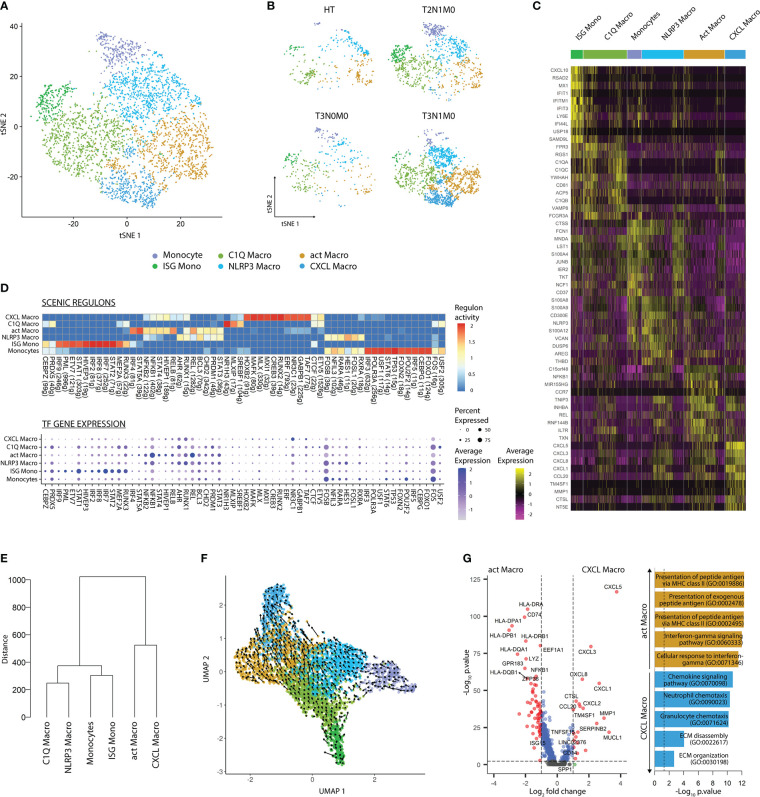
Mono-Mac subsets display tumor-stage specific patterns. **(A)** tSNE plot showing the six transcriptomically unique Mono-Mac clusters across cancer and healthy tissues. **(B)** tSNE plot of Mono-Mac distribution according to TNM classification. **(C)** Heatmap of the top 10 DEG per cluster after DGEA (FC > 1.5, p-value < 0.05). **(D)** Heatmap of the top 10 regulons per cluster colored by regulon activity (upper) and dot plot displaying the gene expression levels of each regulon’s TF per cluster (lower). **(E)** Dendogram of Mono-Mac clusters based on Euclidean distance. **(F)** UMAP plot showing the inferred development trajectory of Mono-Macs using RNA velocity. **(G)** DEG between CXCL and act Macro clusters, and enriched GO pathways obtained after GSEA. DEG, differentially expressed genes; DGEA, differential gene expression analysis; GSEA, gene set enrichment analysis.

Similar to DC lineages, we subsetted Mono-Macs and re-clustered this lineage to address its heterogeneity. Using the SLM clustering algorithm, followed by DGEA, we detected and characterized six transcriptomically unique clusters of Mono-Macs, with different distributions based on TNM classification of TCs (**Figures 3A-C**; **Figures S3A, B** and **Table S6**). Monocytes, expressing high levels of *FCN1*, *CTSS*, and *JUNB*, were highly enriched in blood CD14^+^ and CD16^+^ monocyte as well as MDSC signatures. In turn, ISG Monos featured high levels of IFN inducible genes (*IFIT1*, *IFIT3*, *IFITM*), and a high score of an ISG Mono gene-set. NLRP3 Macros expressed high levels of *NLRP3*, *CD300E*, and *AREG*, and were preferentially enriched in an NLRP3 and MDSC gene-sets. C1Q Macros expressed *C1QA/B/C* genes similarly to MAFB^+^ LC, but uniquely expressed *FCGR3A* and were enriched in a C1Q tumor-associated macrophage (TAM) gene-set. A cluster displaying expression of *IL7R*, *CCR7*, *MIR155HG*, and *CD274* was annotated as activated macrophages (act Macro). Finally, CXCL Macros showed high expression of *CXCL1/3/5*, *SPP1*, and *MMP1* as well as high score of a SPP1 TAM gene-set. Remarkably, signature scores corresponding to M1 and M2 activation states (37) did not distinguished the Mono-Mac clusters, indicating that the M1/M2 dichotomy did not resolve the differences between these (**Figure S3A**). Apart from CXCL Macros, all Mono-Mac clusters were found in HT and TC, regardless of tumor stage. Instead, CXCL Macros were only detected in advanced primary tumors (n=2, T3N1M0, **Figure 3B**).

We evaluated gene regulatory network activity and transcription factor expression in Mono-Mac clusters (**Figure 3D**; **Table S7**). Briefly, the Mono-Mac lineage featured common expression of FOS and FOSB, where monocytes presented the higher regulon activity. In addition, Monocytes and NLRP3 Macros shared preferential expression and regulon activity of HES1, RARA, RXRA, and NFIL. In comparison, ISG Monocytes displayed expression and high regulon activity of IRF7/8 and STAT1, and shared RUNX3 and MEF2A regulon activity with C1Q Macros. Finally, CXCL and act Macros shared expression and activity of certain regulons including STAT4 and HIVP1, but while the former displayed preferential NR3C1 and TAF7 activity and expression, the latter featured higher STAT5A, NFKB1, and REL activity. We compared the transcriptomic similarity between the Mono-Mac clusters by performing hierarchical clustering based on Euclidean distance (**Figure 3E**). Monocytes and ISG monocytes, NLRP3 and C1Q Macros, and CXCL and act Macros, were paired together, respectively.

Next, we projected single cell transcriptomes along with RNA velocity information onto UMAP embeddings to decipher future state of cells (**Figure 3F**). We observed that Monocytes and ISG mono differentiated into NLRP3 and C1Q Macros, which in turn polarized into CXCL and act Macros, respectively. Furthermore, we evaluated transcriptomic changes across the Mono-Mac developmental trajectories by analyzing highly variable genes with dynamic changes in their velocity (**Figure S3C**). We noted that Monocytes, ISG Monos, NLRP3, and C1Q Macros upregulated the expression of *IL1B* and *OLR1*, while the expression of these genes was drastically downregulated upon activation into CXCL and act Macros. Monocytes and NLRP3 Macros featured active transcription of *NLRP3*, *S100A9*, *AQP9*, and *FCN1*. Upon progression into CXCL Macros these upregulated *MMP10* and *CXCL5*. On the contrary, ISG Monos presented active transcription of *ISG20* and *IDO1*, and sequentially upregulated *HLA-DPB1*, *IL4I1*, and *IRF4* as they differentiated into C1Q and act Macros, respectively. Together these results highlighted that there was a core transcriptional program in different stages of the Mono-Mac lineage trajectory.

To gain insights into the two parallel Mono-Mac differentiation processes, we performed pairwise DGEA between act Macro and CXCL Macro clusters followed by GSEA (**Figure 3G**). act Macros featured a higher expression of antigen presentation related transcripts and enrichment of the corresponding pathways. In turn, CXCL Macros were characterized by high expression of chemotactic genes related to granulocyte and neutrophil chemotaxis (*CXCL1*/*3*/*5*/*8*), angiogenesis (*VEGFA*), and genes related to matrix remodeling pathways (*MMP1*, *SPP1*). These results highlighted distinct functions of the terminal macrophage states in the two differentiation pathways present in our dataset.

### 3.4 Validation of myeloid heterogeneity in TC and paired HT

**Figure 4:**
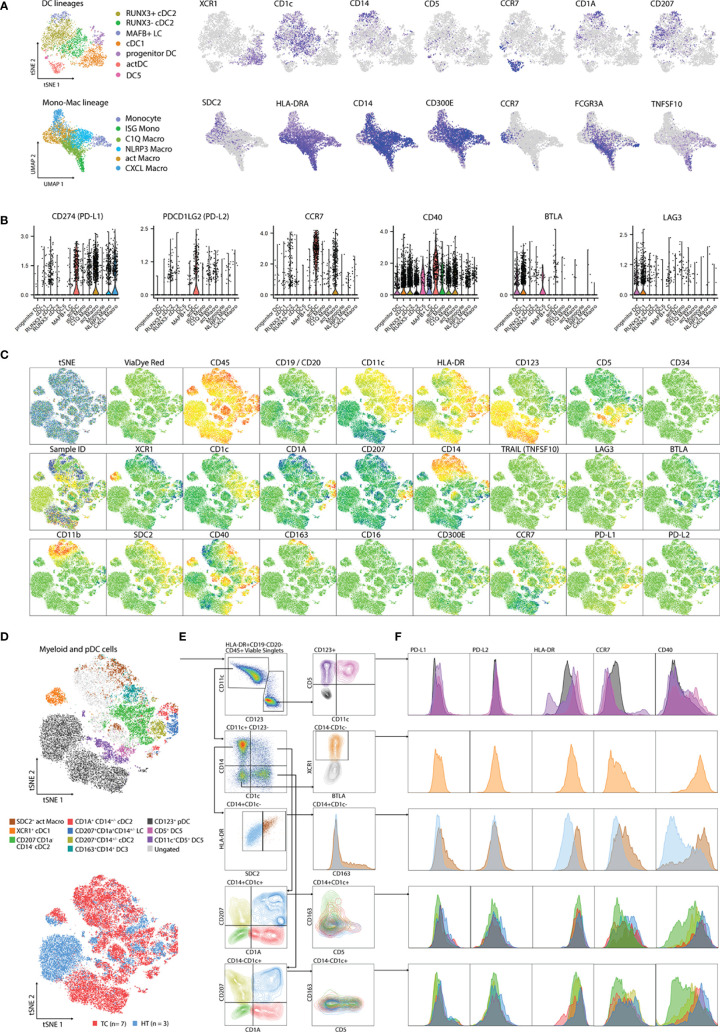
Validation of scRNA-seq clusters using 26-plex flow cytometry. **(A)** Gene expression of cluster-specific markers mapping to cell membrane from scRNA-seq analysis. **(B)** Expression of genes related to APC function from scRNA-seq analysis. **(C)** tSNE plots showing individual marker expression on 45,712 viable CD45^+^HLA-DR^+^CD19^-^CD20^-^ single cells from TC (n=7) and paired HT (n=3). **(D)** tSNE plots showing validated populations and their distribution in TC and HT tissues. **(E)** Gating strategy used to identify scRNA-seq clusters using flow cytometry. **(F)** Expression of genes related to APC function from flow cytometry. DC, Dendritic cell; TC, Tonsillar cancer; HT, Healthy tonsil; pDC, plasmacytoid DC.

Next, we sought to define a flow cytometry antibody panel to validate the myeloid communities detected by scRNA-seq at protein level. Protein targets were selected by performing DGEA analysis across myeloid cell clusters, followed by a filtering step to select cell membrane protein coding transcripts (**Table S8**). We evaluated TC and HT-derived single-cell suspensions using an antibody panel directed towards proteins encoded by genes identified in our scRNA-seq analysis (*XCR1*, *CD1c*, *CD14*, *CD5*, *CCR7*, *CD1A*, *CD207*, *SDC2*, *HLA-DRA*, *CD300E*, *FCGR3A*, and *TNFSF10*) (**Figure 4A**). The panel was complemented with antibodies specific for immune-checkpoints PD-L1, PD-L2, LAG3, and BTLA as well as the co-stimulatory protein CD40, given that their corresponding genes exhibited cluster/lineage specific expression in our scRNA-seq dataset (**Figure 4B**). Antibodies against CD19 and CD20 targeting B-cells, and CD123 targeting plasmacytoid DCs, were included in the panel to increase resolution and compare myeloid cells to other APC populations. We also included other markers in our panel that, despite not being detected in our analysis, have recently been reported to mark novel myeloid populations. CD163 has been reported to mark a CD14^+^ DC3 population possibly distinct from CD14^+^ LC and moDC (30, 31). CCR2 was included in the panel since it is downregulated by monocytes upon differentiation (36, 58), while CD34 was included to detect cDC progenitors (30).

We analyzed ten cryopreserved single cell suspensions, corresponding to biopsies from seven TCs and three paired HTs, by flow cytometry (**Table S1**). After singlet filtering, viable CD45^+^HLA-DR^+^CD19^-^CD20^-^ cells were concatenated and visualized within a tSNE space. We observed a clear signal from all markers apart from CD16, LAG3, TRAIL, BTLA, CD34, and CD300E (**Figure 4C**). We established a gating strategy to define several myeloid populations in human TC and HT and compared their levels of PD-L1, PD-L2, HLA-DR, CCR7, and CD40 (**Figures 4D-F**). We obtained a clear separation of the dataset based on CD11c and CD123, with minimal overlap between the two markers. Careful inspection of the CD123^+^ gate revealed two small CD5^+^ populations corresponding to DC5s, which were distinguishable by CD11c expression, as previously reported in blood (30). Compared to pDCs, DC5s featured higher levels of HLA-DR, CD40, and CCR7. In turn, inspection of the CD11c^+^CD14^+^CD1c^-^ Mono-Mac gate revealed a parallel increase of HLA-DR along the SDC2 signal. SDC2^+^ HLA-DR^high^ corresponded to act Macros according to their co-expression of PD-L1, CD40, and CCR7 at protein level, as observed at RNA level. In addition, this population exclusively expressed CD163 compared to other CD14^+^ Mono-Mac populations. Next, we focused on resolving the heterogeneity of the CD1c^+^ gates. Similar to our scRNA-seq analysis, we observed co-expression of CD1c and CD207 in CD14^+/-^ cDC2s, suggesting that these corresponded to our newly identified RUNX3^+^ cDC2 cluster. In turn, CD1a^+^CD207^+^CD14^+/-^ was identified as LC. Interestingly, we observed a co-expression of CD163 and CD14 at protein level in CD1c^+^CD1A^-^CD207^-^ cells. This indicates that DC3s are present in TC, as previously reported in blood (30, 31) and other HNCs (40), but are indistinguishable from other CD1c^+^ cells by scRNA-seq at our sequencing depth. In turn, CD5 expression inversely correlated with CD14 in CD1c^+^ cells, suggesting that these were *bona fide* cDC2s, as described in blood (31). While all CD1c^+^ populations had some expression of PD-L1, PD-L2, HLA-DR, CCR7, and CD40, CD207^-^CD1A^-^CD1c^+^ cDC2s showed the most immature profile according to CD40 and CCR7 levels. Finally, XCR1^+^ cDC1s were clearly distinguishable by their high CD40 and HLA-DR levels, CCR7 expression, and residual PD-L1 and PD-L2 expression, indicating a mature profile of these population.

To assess transcriptomic changes induced by the TME, we performed DGEA comparing TC and HT myeloid populations from our scRNA-seq dataset (**Figure S4**, **Table S9**). Progenitor DCs in TC expressed higher levels of cDC1 canonical genes (*CLEC9A*, *BATF3*, *XCR1*) and cell cycle genes (*CCND1*, *CAMK2D*), as illustrated by the enrichment in blood cDC1 and cell cycle scores, respectively. In contrast, progenitor DCs in HT featured higher levels of canonical cDC2 genes (*CLEC10A*, *CD1C*) and blood cDC2 gene-set score. On comparing DEG between TC and HT, we observed a common increase of IFN response-related genes (*ISG15*, *ISG20*, *TXN)* in all DC clusters. Conversely, all myeloid populations in TC showed a downregulation of inflammasome-regulation genes *MTRNR2L12*, *DNASE1L3*, and *LGALS2* as well as MHC-II coding genes.

### 3.5 cDC1s and activated DCs and macrophages correlate with increased survival in HNC patients

We sought to assess the presence of myeloid populations described here in TC and HT, in other HNCs. To this end, we accessed publicly available scRNA-seq data (41), subset myeloid cells and integrated them using our in-house dataset as reference. We observed that all clusters identified in our in-house TC dataset were also present in other subtypes of HNC, although in varying frequencies (**Figure S5A**). RUNX3^-^ cDC2s were only marginally present in HNC subtypes (4 predicted cells), indicating that this cell state was specific to healthy tissue as described above (**Figure 2A**) thus validating our prediction. Surprisingly, the diversity of tumor-infiltrating myeloid cells was similar between HPV^+^ and HPV^-^ tumors (**Figure S5B**).

**Figure 5:**
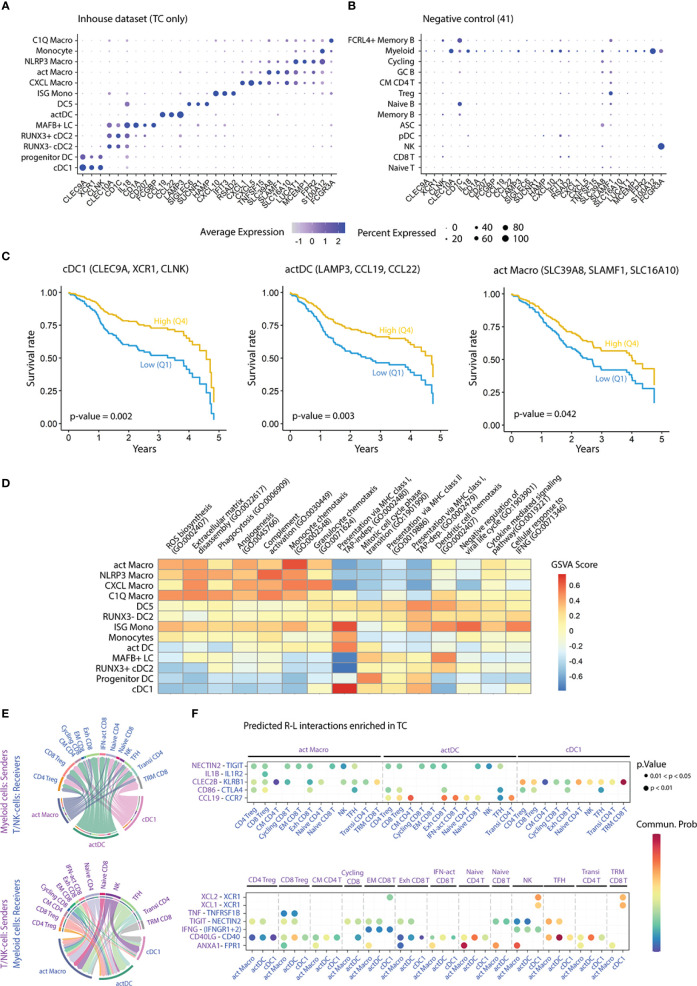
Impact of myeloid populations on patient survival, as well as functions and interactions thereof. **(A)** Dot plot displaying cluster-specific signature genes in the myeloid dataset and **(B)** in a publicly available scRNA-seq dataset (41). **(C)** Survival curves showing five-year overall survival rates for the indicated gene-sets, from a publicly available bulk RNA-seq dataset of HNC patients (n=529) (52). Comparison of survival probability was performed using log-rank test and Cox proportional-hazards model. Years: years since diagnosis. **(D)** Heatmap colored by GSVA score of enriched GO pathways in the myeloid clusters (adjusted p-value < 0.05). **(E)** Chord diagrams displaying the interaction between cell pairs. Interaction strength is presented as the weight of the edges, and the receiver cell cluster is indicated by the apex of the arrow. **(F)** Bubble plot showing statistically significant receptor-ligand (R-L) coding gene pairs in TC between myeloid cell subsets and T/NK- cells from a publicly available dataset (41). GSVA, gene set variation analysis; FC, fold change; p, p-value; CM, central memory; EM, effector memory; Exh, exhausted; IFN-act, IFN-activated; NK, natural killer; TFH, T follicular helper; Transi, transitional; TRM, tissue-resident memory.

To investigate the prognostic impact of myeloid populations in HNC, we defined gene signatures that enabled us to infer the relative abundance of myeloid populations within bulk HNC RNA-seq. We performed DGEA across all myeloid clusters that were isolated from TC, obtaining a total of 998 DEG (FC ≥ 2, p-value ≤ 0.01, **Table S8**). Then, we filtered our candidate genes using a publicly available scRNA-seq HNC dataset (41), to select cluster-specific genes that were not expressed by other tumor infiltrating-leukocytes (**Figures 5A, B**). We obtained 3-gene transcriptomic signatures for all our clusters except progenitor DCs, monocytes, and C1Q Macros. We further investigated the impact of these 3-gene signatures within bulk RNA-seq transcriptomes of HNC from the TCGA (52). We applied Cox proportional-hazards regression to compare the survival rate of high (Q4) and low (Q1) scoring quartiles of each gene signature (**Figure 5C**, **Table 1**). We found that high scores of cDC1, actDC, and act Macro gene-sets correlated with increased five-year survival in HNC, independently of age and sex. Of note, high score of the cDC1 signature was also positively correlated to survival when TC patients were assessed separately (**Figure S5C**).

**Table 1:** Adjusted hazard ratios (HR) comparing the five-year overall survival rate between HNC patients with high (Q4) and low (Q1) cluster-specific gene-set scores.

Cell-specific gene signature	Univariate model			multivariate model		
	HR (95% CI)	p-value	N	HR (95% CI)	p-value	N
**cDC1**	**0.48 (0.3-0.77)**	**0.0023**	**225**	**0.47 (0.29-0.76)**	**0.0022**	**225**
cDC2-like	0.94 (0.63-1.39)	0.767	229	0.93 (0.61-1.4)	0.738	229
LC	0.81 (0.54-1.21	0.316	226	0.93 (0.61-1.41)	0.746	226
**actDC**	**0.51 (0.33-0.78)**	**0.0023**	**227**	**0.52 (0.34-0.81)**	**0.0036**	**227**
DC5	0.94 (0.61-1.44)	0.788	222	0.93 (0.61-1.43)	0.767	222
ISG Mono	0.85 (0.56-1.28)	0.453	224	0.77 (0.5-1.17)	0.234	224
CXCL Macro	1.19 (0.79-1.79)	0.38	226	1.13 (0.75-1.7)	0.557	226
**act Macro**	0.68 (0.45-1.03)	0.074	225	**0.64 (0.42-0.95)**	**0.042**	**225**
NLRP3 Macro	1.01 (0.67-1.5)	0.96	229	0.97 (0.65-1.45)	0.894	229

The multivariate HR model was corrected by gender and age at diagnosis. HR, hazard-ratio; CI, confidence interval; N, number of patients. Statistically significant gene-signatures are indicated in bold.

Next, we aimed at elucidating mechanisms by which cDC1s, actDCs, and act Macros impacted survival. Pathway enrichment analysis (GO bioprocesses 2018 (56), adjusted p-value < 0.05) revealed subset specific functions within the myeloid compartment in TC (**Table S10**). Highly scoring pathways related to myeloid cell function, such as antigen-presentation and chemotaxis, were subjected to GSVA score transformation and compared across clusters (**Figure 5D**). Overall, we found that all DCs, but also ISG monos, featured higher scores for antigen presentation pathways compared to macrophages. Specifically, the cDC1 cluster displayed the highest score in pathways related to cross-presentation *via* MHC-I, followed by ISG monos, DC5s, and actDCs. In contrast, all DC clusters presented similar scores in presentation *via* MHC-II, which was higher compared to Mono-Mac clusters. In turn, macrophage clusters were characterized by higher expression of genes related to chemotaxis, phagocytosis, ECM disassembly, angiogenesis, and complement pathway. Notably, ISG monocytes were preferentially enriched in cellular response to IFNG and negative regulation of viral life cycle, suggesting a TME-specific polarization of this cluster.

To investigate which leukocytes that interacted with cDC1s, actDCs, and act Macros, we accessed a publicly available HNC scRNA-seq dataset (41). After annotating single cells from TC and HT based on canonical marker expression, we integrated all leukocytes of the external dataset and our in-house dataset (**Figure S5D**). Following normalization, we performed receptor ligand analysis to identify major cell-cell interactions in TC as well as receptor-ligand gene pairs overexpressed in TC as compared to HT (**Table S11**). We focused our analysis on the interaction of myeloid cells with T/NK-cell subsets, because of the high abundance of the latter in the TME (**Figures 5E, F**). cDC1s featured higher signaling strength with NK and tissue resident memory (TRM) CD8 T-cells, followed by Treg subsets, as compared to other T/NK-cells. Furthermore, cDC1s interacted with TRM CD8 T-cells, effector-memory (EM) CD8 T-cells, and NK-cells *via* XCL1/XCL2-XCR1 as well as CLEC2B-KLRB1 axes. Based on the predicted signaling strength, actDCs and act Macros interactions were more diverse than for cDC1s. Both actDCs and act Macros were predicted to interact with several T/NK-subsets *via* NECTIN2-TIGIT. However, while actDCs were uniquely predicted to interact with T/NK-subsets *via* CCL19-CCR7, act Macros displayed a high probability of interacting *via* ANXA1-FPR1. The interaction *via* CD40-CD40LG axis was predicted in several myeloid and T/NK-subset combinations, but it was highest between actDC and T-follicular helper (TFH), naïve, and transitional CD4 T-cells. Finally, the common predicted interactions of cDC1, actDC, and act Macro clusters in TC included: IFNG-IFNGR1/2 with NK and EM CD8 T-cells, as well as CD86-CTLA4 with Tregs, exhausted CD8 T-cells, and TFH cells.

## 4 Discussion

HPV-driven TC is a subset of HNC characterized by type 1 T-cell inflammatory responses (40). Effective cellular immune responses are complex phenomena involving an interplay of different cell types. Myeloid cells represent a particularly interesting entity due to their marked ability to prime T-cells and their wide diversity (30). Motivated by the expansion of the CD13^+^HLA-DR^+^ population in HPV^+^ TC compared to paired HT, we sought to unbiasedly assess their heterogeneity. Here, we present a high-resolution characterization of TC infiltrating and lymphoid tissue resident myeloid cells. We generated a scRNA-seq dataset of 9,505 CD45^+^CD13^+^HLA-DR^+^ myeloid cells from TC biopsies and paired HT from treatment naïve patients using 10X Genomics. This dataset represents a tool for understanding myeloid identity dynamics in the tumor as well as steady state lymphoid tissue settings. By combining transcriptome profiling, gene signature scoring, regulon activity assessment, and RNA velocity, we obtained a dynamic snapshot that illustrates the interrelationship of different myeloid communities. In addition, pathway enrichment, R-L, and survival analyses of in-house and publicly available HNC datasets yielded information on how myeloid communities impact survival and on the underlying molecular mechanisms and cell-cell interactions.

DC identity has recently been re-assessed in blood (30), lymphoid organs (32, 33), and several types of cancer (11, 28, 35). Here we identified seven DC populations corresponding to four different lineages and diverse cell states. We observed that the cDC1 cluster was consistently homogeneous through tissue types, and that its identity was dictated by the IRF8 and STAT2 regulons, as described previously (59, 60). Interestingly, cDC1s also featured high regulon activity and expression of *SMARCA5*. This TF has recently been characterized in viral DNA sensing and induction of IFN pathways, enabling DCs to prime CTL responses with a marked IFN-γ and IL-12 production (61, 62). In this context, our in-house generated cDC1 signature highlights a positive impact of cDC1s on HNC patient survival, and for TC patients when assessed separately. Pathway enrichment analysis evidenced a strong expression of genes related to MHC-I-mediated cross-presentation, which is a well characterized trait of the cDC1 lineage (16). Complementing the hypothesis that cDC1s migrate in the tumor through the XCL1/2 – XCR1 axis (15), we show that, besides for NK-cells, *XCL1*/*XCL2* were also expressed by a subpopulation of *ENTPD1^+^ ITGAE^+^* CD8^+^ T-cells. In fact, the interaction strength between these CD8^+^ T-cells and cDC1s was also mediated by the CLEC2B-KLRB1 axis. All in all, our data suggest a key interaction between cDC1 and a small subpopulation of TRM CD8^+^ T-cells that has been reported to exert a tumor-reactive function in the TME of several cancer types including HNC, but also ovarian, lung, and colon cancer (63). Interestingly, RNA velocity analysis indicated that the cDC1 population stems from a cycling progenitor DC-population to later mature into CCR7^+^ LAMP3^+^ actDC, suggesting that cDC1s acquire a migratory potential in the TME. By comparing the signature scores of the progenitor DC population in TC and HT, we observed a marked increase in cell cycle genes and a preferential blood cDC1 signature enrichment in TC’s progenitor DCs. This indicates that progenitor DCs infiltrating TC are mainly pre-committed to a cDC lineage as is the case in blood (64). In addition, it suggests that, in the context of virally driven TC, there is a high production rate of cDC, and that these are pre-committed to cDC1, to the detriment of cDC2. This hypothesis is supported by the overall increase in myeloid APC frequency in TC, including cDC1s, while cDC2 populations were similarly abundant, as estimated both in scRNA-seq analysis and by flow cytometry. Brown et al., previously reported the presence of mitotic pre-cDCs in the spleen (33). To our knowledge, our study is the first report of cycling pre-cDCs in cancer, shifting the balance towards the cDC1 lineage.

Similar to what has been reported for blood (30) and spleen (33), DC2-like cells displayed higher heterogeneity compared to cDC1s both at RNA and protein level. Our dataset recapitulates transcriptomically distinct clusters with common *CEBPB* regulon activity (65). In scRNA-seq, MAFB^+^ LC acted as a source of DC2-like cells in TC, while RUNX3^-^ cDC2s were the unique source in HT, as seen in the RNA velocity analysis. MAFB^+^ LC uniquely exhibited MAFB and RXRA regulon activity, as well as expression of CD207, CD14, CD1A and CD1C at gene and protein level. Furthermore, these featured high scores for skin LC and tissue cDC2 gene sets (35), and no enrichment in a DC3 gene-signature, indicating that these cells did not correspond to the recently characterized DC3 subset in blood (30, 66). In fact, our flow cytometry results showed that LC and DC3 are two distinct entities judging by the lack of CD163 co-expression with CD207 and CD1a. In addition, some MAFB^+^ LC exhibited high scores of G2 and M cell cycle phases, indicating that these cells had self-renewal capacity in tonsillar tissue, as they do in skin (38, 67). MAFB^+^ LCs featured high RUNX3 upregulation, and downregulated the activity of the MAFB regulon on their differentiation into RUNX3^+^ cDC2s. In line with our results, previous studies have reported expression of *MAFB* and *RUNX3* during LC differentiation in mouse oral mucosa and skin (68–70). In contrast, RUNX3^-^ cDC2s most likely represented *bona-fide* lymphoid organ resident cDC2s, as judged by their exclusive presence in HT, absence in TC and other HNC subtypes, and the lack of monocyte lineage markers and regulons at RNA and protein level. We observed that cDC2s upregulated RUNX3 along with CD207 and interferon response genes *ISG15*, *IFI30*, and *IFITM3* in TC. We hypothesize that, in the TME, cDC2s are subjected to IFN-I dependent maturation, potentially *via* TLR stimulation as it occurs upon viral or poly I:C stimulation (71, 72). Consistent with our observation at RNA level, CD207^+^CD1a^-^ cDC2s featured a more mature profile at protein level as compared to their CD207^-^ counterparts. Upon maturation into actDCs, DC2-like cells downregulated the expression of *RUNX3*, which is a known repressor of *CCR7* expression and cDC2 maturation (73). Collectively, our RNA-velocity analysis showed that both cDC2 and LC matured into actDC gaining *CCR7* expression, as reported previously by Bennewies et al. and Reynolds et al. (14, 39). Compared to other myeloid populations, MAFB^+^ LC expressed high levels of *TGFB*, which has been shown to inhibit CCR7 expression by DC *in vitro* and cause intra-tumoral retention of act DCs in mouse models (74, 75). Survival analysis based on signature scoring of the TCGA dataset did not suggest a potential contribution to HNC or TC patient survival of either of the three DC2-like populations. However, DC2-like cells, as well as cDC1s, converged into an actDC gene-program governed by *RELB* (76) and *IRF1*/*2* (77), which was associated with an increase in survival, as assessed by gene-signature scoring. Pathway enrichment analysis suggested that actDCs perform antigen presentation *via* MHC-I and II in the TME and interact with diverse T-cell populations. Interaction through the CCL19-CCR7 axis is indicative of active T-cell recruitment into TC’s TME (78, 79), while the CD40-CD40LG axis suggests a positive feedback-loop between type-1 T-cell polarization and DC maturation (80). The synergy between sequential ligation of IFNAR1/2 and CD40 on the surface of DC is known to boost DC maturation (81). Thus, targeting myeloid cells through CD40 in an IFN-rich microenvironment, as the one of HPV^+^ TC, may represent a viable therapeutic alternative to immune-checkpoint blockade strategies.

We also detected a rare DC cluster expressing *ILR3A, SIGLEC6*, and *AXL* in close transcriptomic proximity to DC2-like cells, which has been recently termed DC5 (30), AS DC (33), and transitional DC (82). As reported previously, DC5 identity was linked to TCF4 and RUNX2 regulon activity (33, 82). As for cDC1 and DC2-like cells, DC5s expressed high levels of the transcription factor *ZNF366*, which is a known repressor of the pDC developmental program, thus suggesting a myeloid origin of this population, congruent with our myeloid focused sorting strategy (83). DC5s have been reported to differentiate into cDC2-like cells *in vitro* (30): however, despite detecting CD11c expression on some TC derived DC5s by FC, we did not observe directionality towards DC2-like cells in RNA-velocity analysis. Together these results suggest a limited polarization of DC5s into cDC2 in the TME. Nonetheless, DC5s featured higher HLA-DR, CD40, and CCR7 protein expression compared to plasmacytoid DCs. Hence, specific functions of this rare subpopulation remain to be determined. Additionally, we detected a granulocyte lineage within the CD13^+^HLA-DR^+^ population, corresponding to mast cells with moderate expression of MHC-II related transcripts. Some studies have reported that granulocytes gain MHC-II expression during inflammation, highlighting their plasticity within an appropriate niche (84). Specifically, mast cells gain MHC-II expression upon exposure to IFN-γ, IL-4, and GM-CSF *in vitro*, although their specific ability to present antigens and activate T-cells *in vivo* is under debate (85).

We have previously demonstrated the expansion of the Mono-Mac lineage in HPV^+^ TC compared to paired HT (86), which indicates an active recruitment of monocytes during carcinogenesis. In this study, we characterized a parallel differentiation process that stemmed from different monocyte polarization events, using RNA velocity analysis. Our results challenge the *in vitro* M1/M2 paradigm, similarly to other scRNA-seq studies (11, 35). Monocytes expressing high levels of *FOS* and *FOSB* differentiated sequentially into NLRP3 and CXCL Macros. Of note, monocytes and NLRP3 Macros displayed the highest level of MDSC gene signature, which have been reported to differentiate into tumor-associated macrophages in tumor mice models (27). However, we did not observe key immunosuppressive features of MDSCs such as expression of *IL10* and downregulation of *TNF* in our analysis (87). Together these observations suggest that monocytes and NLRP3 macrophages have limited immunosuppressive capacity in HPV^+^ TC. Nonetheless, NLRP3 macrophages differentiated into CXCL Macros, which were only found in high tumor stages and featured the highest activity of the hypoxia-related regulons HIF1A and CREB and their target genes downstream including *VEGFA* and *CXCL1/3/5/8* (88, 89). Our data suggest that in high tumor stages CXCL macrophages develop in the presence of hypoxic conditions, and trigger angiogenesis and recruitment of polymorphonuclear-MDSC possibly *via* CXCR2 and ACKR1 (90, 91). Indeed, several studies have reported accumulation of polymorphonuclear-MDSCs in high tumor stages (92) and an association with poor prognosis for HNC patients (93). The second branch of the Mono-Mac lineage sourced from ISG Monos, which uniquely featured activity and expression of the IFNα master regulator IRF7 (94), possibly through trimerization of STAT1/2 and IRF9 (95). ISG Monos expressed *TRAIL*, which may be involved in tumoricidal mechanisms, as described *in vitro* for IFNα-polarized monocytes (96). In addition, ISG Monos expressed the highest levels of *CXCL9/10/11*, to potentially recruit CD8 T-cell in the TME through *CXCR3*, and displayed enrichment of TAP-independent antigen presentation *via* the MHC-I pathway. Classically, these traits have been attributed to cDC1s (97), but they might also be governed by *IRF8* in human monocytes (98, 99) as described in mice (100, 101). Interestingly, ISG Monos sequentially differentiated into C1Q and act Macros, the latter of which had a moderate positive impact on HNC patient survival. Like actDCs, act Macros TF expression profile was associated with RELB and NFKB1/2, but the expression levels and gene-network activity were not as pronounced. We further validated the presence of act Macros by FC, proposing SDC2, CD163, CCR7, PD-L1, and CD40 as phenotypic surface markers. Mulder et al. described similar macrophage populations in lung, liver, and colon cancer: however, they linked their activity to recruitment of Treg and attenuation of T-cell responses *via* PDL1 and PDL2, features that we did not predict in our analysis (35). The function and location of CCR7^+^ act Macros may deserve further attention to elucidate if this population can migrate to draining lymph nodes, or if it is rather involved in intra-tumoral gradients of CCL19/21 such as those derived from tertiary lymphoid structures in HPV^+^ tumors (79). act Macros show marker similarity with other reported populations such as human tonsillar macrophages and CD169^+^ subcapsular sinus macrophages (32, 102). Hence, act Macros could be involved in the induction of TFH polarization (32) and the transfer of antigens to cDC1, favoring cross-presentation to CD8^+^ T-cells as described in draining lymph nodes and spleen (102, 103).

In summary, we have provided a comprehensive characterization of myeloid cell identity and dynamics in HPV^+^ TC and HT and validated our findings using 26-plex flow cytometry. Consistent with the idea that HPV^+^ TC is enriched in type-1 immune responses, we have characterized distinct polarization states of DC and Mono-Mac lineages, highlighting the impact of a type-I/II IFN-rich TME on myeloid cells. Among all populations, cDC1s stand as an ideal therapeutic target due to the biased production of their precursors, their increased abundance and capacity to mature in the tumor lesion, and their positive impact on TC and HNC patient survival. Our data support the conceptualization of targeted-therapeutic strategies to modulate intra-tumoral cDC1 activity, and delineate myeloid cell profiles that potentially may be used for patient stratification and treatment selection.

## Data availability statement

The original contributions presented in the study are publicly available. This data can be found here: https://www.ncbi.nlm.nih.gov/geo/query/acc.cgi?acc=GSE219210, Accession number; GSE219210.

A full collection of the pipeline that replicates the data analysis outlined in this article will also be made available as an R script repository on Github upon request.

## Ethics statement

The studies involving human participants were reviewed and approved by Swedish Ethical Review Authority (ref. no. 2017/580). The patients/participants provided their written informed consent to participate in this study.

## Author contributions

Conceptualization, DJ, LG, and ML. Experiments, DJ, AS and CA. Data analysis, DJ and AA. Data visualization, DJ. Providing human samples, SS, DA, and LG. Supervision LG and ML. Writing—original draft, DJ, LG and ML. Writing—review and editing, all authors. All authors contributed to the article and approved the submitted version.
