# Supplementary material for: Single-cell analysis of myeloid cells in HPV+ tonsillar cancer
Source: Front Immunol. 2023 Jan 19;13:1087843. doi: 10.3389/fimmu.2022.1087843 (PMC9893928; doi:10.3389/fimmu.2022.1087843)
Supplement: Supplementary file 1 [file DataSheet_1.docx]

Supplementary Material

# Supplementary tables

**Table S1.** Patient details. FC: Flow cytometry. M: Male. F: Female.

| **Patient ID** | **Sex** | **HPV status** | **TNM** | **Stage** | **Smoking status** | **Part of study** | **Paired HT** |
| --- | --- | --- | --- | --- | --- | --- | --- |
| TC1 | F | P16+ | T2N1M0 | I | Non- smoker | scRNA-seq and 13-plex FC | - |
| TC2 | M | P16+ | T2N1M0 | I | Non- smoker | scRNA-seq and 13-plex FC | - |
| TC3 | M | P16+ | T3N1M0 | II | Non- smoker | scRNA-seq and 13-plex FC | 13-plex FC |
| TC4 | M | P16+ | T3N1M0 | II | Non- smoker | scRNA-seq and 13-plex FC | - |
| TC5 | M | P16+ | T3N0M0 | II | Ex-smoker | scRNA-seq and 13-plex FC | scRNA-seq and 13-plex FC |
| TC6 | F | P16+ | T2N1M0 | I | Smoker | 13-plex FC | 13-plex FC |
| TC7 | F | P16+ | T2N0M0 | I | Non- smoker | 13-plex FC | 13-plex FC |
| TC8 | M | P16+ | T2N1M0 | I | Non- smoker | 13-plex FC | 13-plex FC |
| TC9 | M | P16+ | T2N2M0 | II | Non- smoker | 26-plex spectral FC | 26-plex spectral FC |
| TC10 | F | P16+ | T4N1M0 | III | Ex-smoker | 26-plex spectral FC | - |
| TC11 | M | P16+ | T2N1M0 | I | Non- smoker | 26-plex spectral FC | - |
| TC12 | M | P16+ | T2N2M1 | IV | Non- smoker | 26-plex spectral FC | 26-plex spectral FC |
| TC13 | F | P16+ | T2N1M0 | I | Ex-smoker | 26-plex spectral FC | - |
| TC14 | M | P16+ | T3N0M0 | II | Non- smoker | 26-plex spectral FC | 26-plex spectral FC |
| TC15 | M | P16+ | T4N2M0 | III | Ex-smoker | 26-plex spectral FC | - |

**Table S2**. Antibodies used in the sorting strategy, 13-plex flow cytometry panel (related to **Figure 1A** and **Figure S1A-B)**.

| **Fluorochrome** | **Antigen** | **Clone** | **Provider** | **Identifier** |
| --- | --- | --- | --- | --- |
| FVS620 | Viability | - | BD | 564996 |
| APC-H7 | CD45 | 2D1 | BD | 560178 |
| APC | CD14 | Tuk4 | Miltenyi | 130-113-147 |
| PE | CD207 | DCGM4 | Beckman Coulter | IM3577 |
| PerCpCy5.5 | CD3 | UCHT1 | BD | 560835 |
| PerCpCy5.5 | CD19 | HIB19 | BD | 561295 |
| PerCpCy5.5 | CD20 | 2H7 | BD | 560736 |
| PerCpCy5.5 | CD56 | B159 | BD | 560842 |
| FITC | CD11b | ICRF44 | BioLegend | 301330 |
| BV786 | CD16 | 3G8 | BD | 563690 |
| BV421 | CD11c | B-ly6 | BD | 562561 |
| BV711 | HLA-DR | G46-6 | BD | 563696 |
| BV605 | CD141 | 1A4 | BD | 740421 |
| BV510 | CD1a | HI149 | BD | 563481 |
| PE-Cy-7 | CD13 | WM15 | BD | 561599 |
| AF700 | CD1c | L161 | Biolegend | 331530 |

**Table S3.** Antibodies used in the spectral 26-plex flow cytometry panel (related to **Figure 4C-F**).

| **Fluorophore** | **Antigen** | **Clone** | **Provider** | **Identifier** |
| --- | --- | --- | --- | --- |
| ViaDye Red | Viability | - | Cytek Biosciences | SKUR7-60008 |
| APC-FIre810 | HLA-DR | L243 | Biolegend | 307674 |
| BV421 | XCR1 | S15046E | Biolegend | 372610 |
| cFluor B548 | CD14 | 63D3 | Cytek Biosciences | SKU R7-20116 |
| BV510 | CD1a | HI149 | BD | 563481 |
| BV785 | C1c | L161 | Biolegend | 331544 |
| PerCP Cy5.5 | CD16 | 3G8 | BD | 560717 |
| PE | CD207 | DCGM4 | Beckman coulter | IM3577 |
| APCR700 | CCR7 | 2-L1-A | BD | 566766 |
| PE-Cy7 | LAG3 | 3DS223H | Thermo fisher | 25-2239-42 |
| BV711 | CD11c | B-ly6 | BD | 563130 |
| APC | SDC2 | 305515 | RnD | FAB2965A |
| BV650 | CD11b | ICRF44 | BD | 740566 |
| BB515 | CD40 | 5C3 | BD | 565258 |
| PerCPeFluor710 | PDL1 | MIH1 | Thermo fisher | 46-5983-42 |
| PE-Cy5 | BTLA | MIH26 | Biolegend | 344506 |
| APC Vio770 | CD300E | IREM-2 | Miltenyi | 130-101-774 |
| eFluor450 | CD19 | HIB19 | Thermo fisher | 48-0199-42 |
| eFluor450 | CD20 | 2H7 | Thermo fisher | 48-0209-42 |
| PE/Dazzle594 | CD163 | GHI/61 | Biolegend | 333624 |
| BV570 | CD45 | HI30 | Biolegend | 304034 |
| cFluorR720 | CD123 | 6H6 | Cytek Biosciences | SKU R7-20013 |
| Alexa Fluor 647 | CD5 | UCHT2 | Biolegend | 300616 |
| BV480 | CCR2 | K036C2 | Biolegend | 357202 |
| BV750 | PDL2 | MIH18 | BD | 747297 |
| BV605 | TRAIL | RIK-2 | BD | 743720 |

# Supplementary figures

#
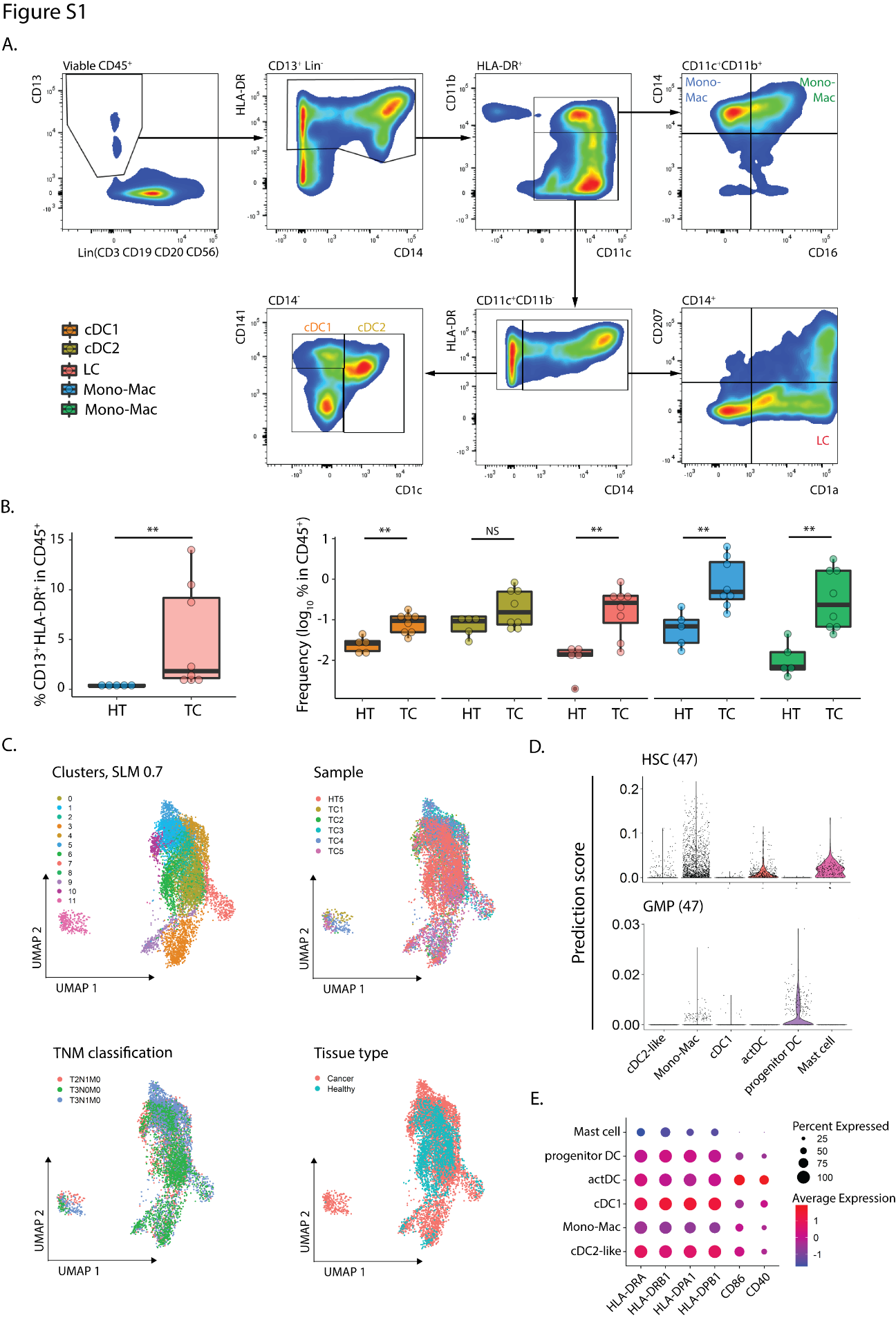


**Figure S1. Related to Figure 1.** (**A**) Gating strategy used to delineate myeloid diversity in TC and HT by flow cytometry. The following populations were identified: cDC1 (CD11b^-^CD14^-^CD11c^+^CD141^+^), cDC2 (CD11b^-^CD14^-^CD11c^+^CD1c^+^), LC (CD11b^-^CD14^+^CD11c^+^CD1a^+^CD207^+/-^), and Mono-Mac (CD11b^-^CD14^+^CD11c^+^CD16^-^ plus CD11b^-^CD14^+^CD11c^+^CD16^+^). (**B**) Box plots displaying frequencies of myeloid populations identified in (A). (**C**) UMAP visualization displaying clusters calculated by SLM 0.7, sample of origin, TNM classification, and tissue type. (**D**) Prediction scores of BM hematopoietic stem-cell and granulocyte-monocyte progenitor obtained from HCA (Human Cell Atlas) (9). (**E**) Dot plot displaying expression of MHC-II related transcripts, *CD86,* and *CD40* across myeloid populations. LC: Langerhans cell; HSC: Hematopoietic stem cell; GMP: Granulocyte-monocyte progenitor.


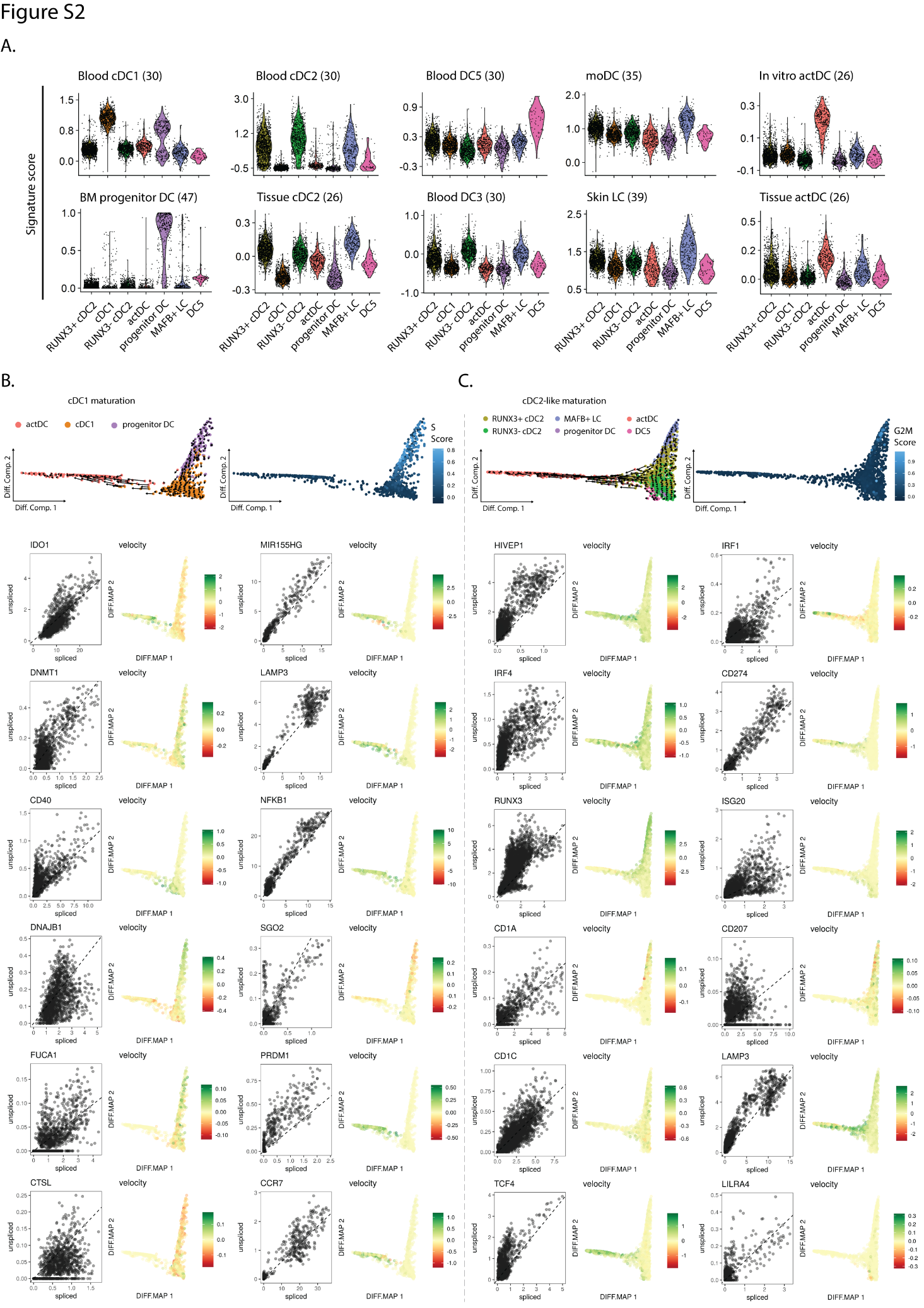


**Figure S2. Related to Figure 2**. (**A**) Gene-set enrichment scores for indicated gene-signatures. (**B**) Global and gene-specific RNA velocity and S scores in cDC1 maturation. (**C**) Global and gene-specific RNA velocity and G2M scores in cDC2-like maturation. G2M and S scores represent the relative expression of cell cycle phase genes in a cell.


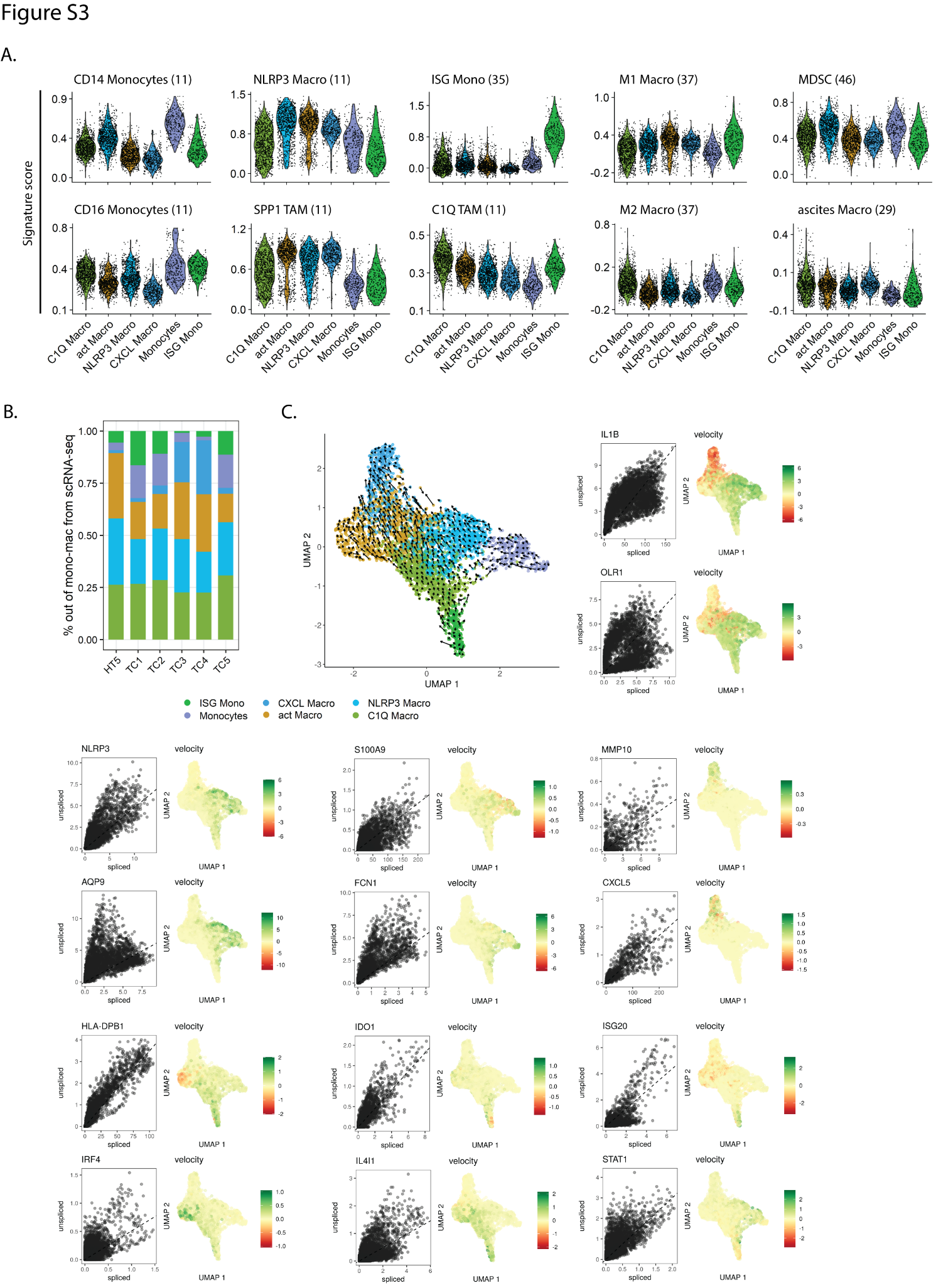


**Figure S3. Related to Figure 3.** (**A**) Gene-set enrichment scores for indicated gene-signatures. (**B**) contingency bar plot displaying the frequency of Mono-Mac populations across tissue of origin. (**C**) Global and gene-specific RNA velocity of Mono-Mac lineage differentiation pathway.


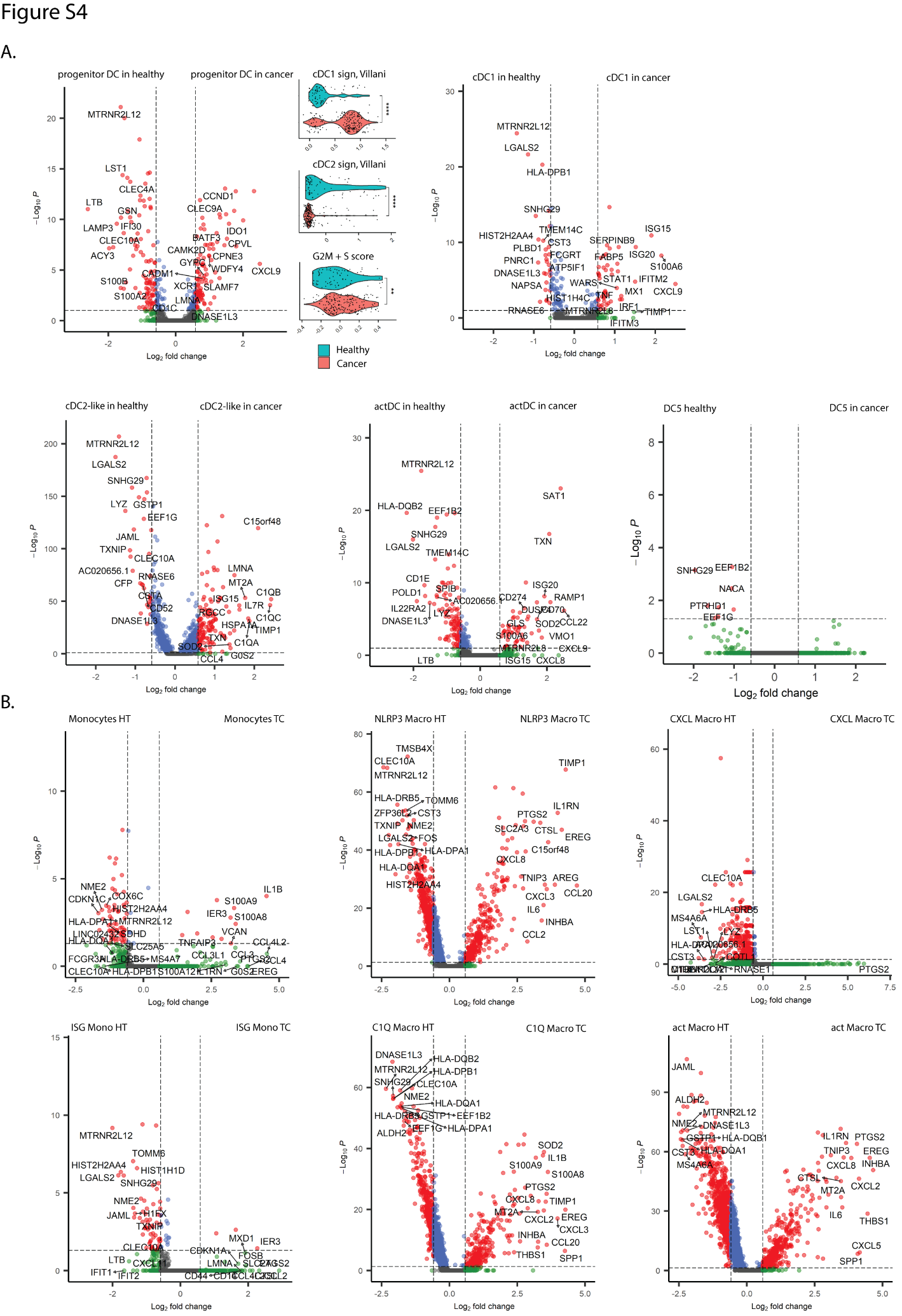


**Figure S4. Related to Figure 4.** (**A**) Volcano plots displaying DEG between TC and HT of indicated DC populations (FC > 1.5, p-value < 0.01). (**B**) Volcano plots displaying DEG between TC and HT of indicated Mono-Mac populations (FC > 1.5, p-value < 0.01). DEG: Differentially expressed genes.


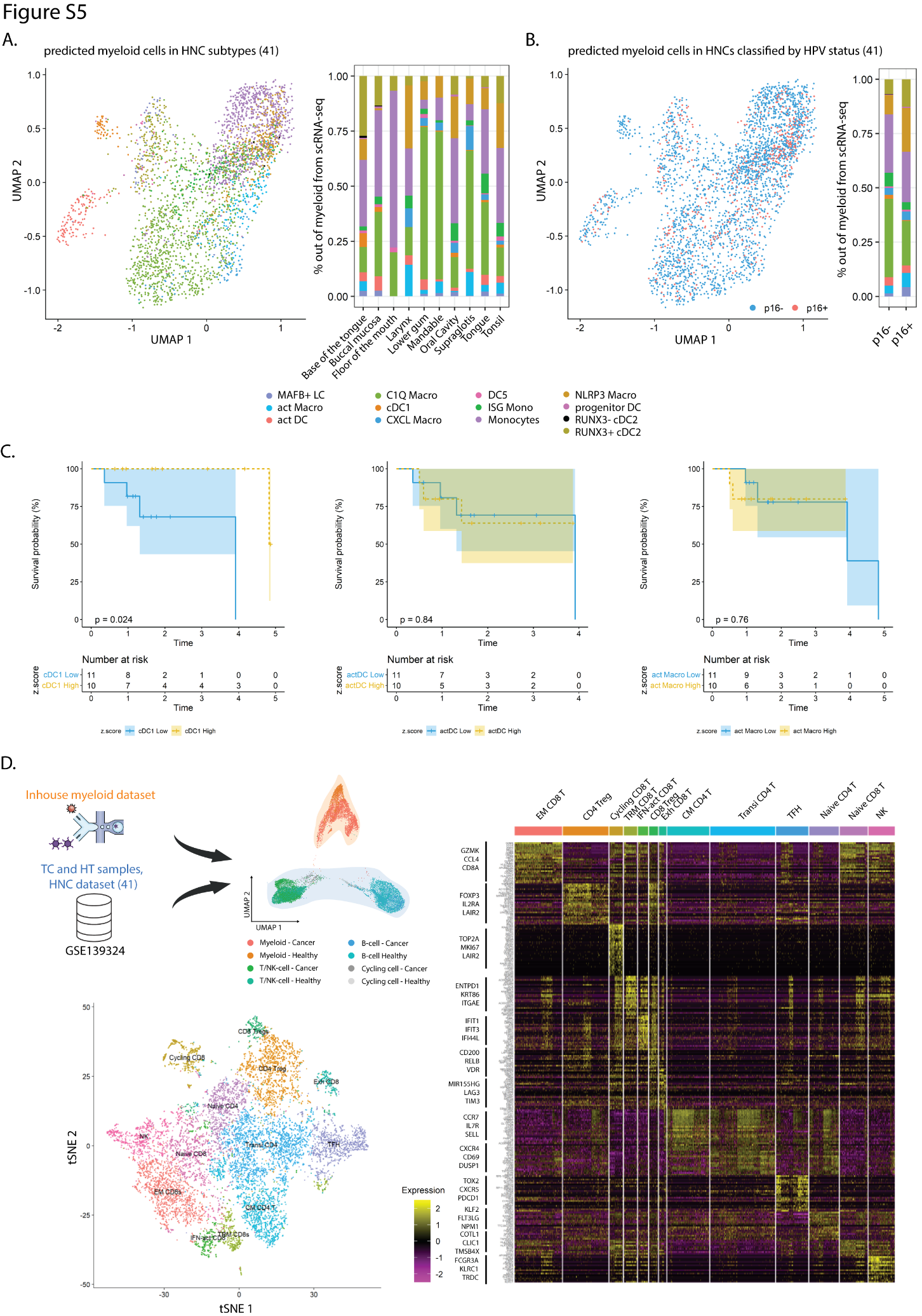


**Figure S5. Related to Figure 5.** (**A**) UMAP plot and contingency bar plot displaying myeloid populations and frequencies from a publicly available cohort of HNC (n=22) (41). (**B**) UMAP plot and contingency bar plot displaying the status of the HNC patient cohort and myeloid frequencies based on HPV status. HPV status was assessed by the detection of p16 by immunohistochemistry. (**C**) Survival curves showing 5-year overall survival rates for the indicated gene-sets, from a publicly available bulk RNA-seq dataset of TC patients (n=40). Comparison of survival probability was performed using log-rank test. Time: Years since diagnosis. (**D**) Strategy to perform receptor ligand analysis by re-analyzing a publicly available TC scRNAseq dataset (41). UMAP displaying integrated T/NK-cell, B-cell, and inhouse myeloid lineages (top left) as well as tSNE displaying T/NK-cell lineage subsets (bottom left) and top 25 DEG per T/NK-cell cluster (right).

# Other supplementary materials for this manuscript include the following

**Movie S1** (separate file). 3D UMAP plot displaying DC clusters and the expression of *CLEC9A*, *CLEC10A,* and *LAMP3*.

**Table S1** - DEG across DC clusters (Figure 2C). (Separate .csv file).

**Table S2** - Inferred regulon activity in DC clusters (Figure 2D). (Separate .csv file).

**Table S3** - DEG across Mono-Mac clusters (Figure 3C). (Separate .csv file).

**Table S4** - Inferred regulon activity in Mono-Mac clusters (Figure 3D). (Separate .csv file).

**Table S5** - DGEA across all myeloid clusters in TC – all genes and membrane mapping genes. (Separate .xlsx file).

**Table S6** - Pairwise DGEA comparing all myeloid clusters in TC vs HT (Figure S4). (Separate .xlsx file).

**Table S7** - Pathway enrichment analysis of all myeloid clusters (Figure 5D). (Separate .xlsx file).

**Table S8 -** Predicted RL interactions across all myeloid clusters and other leukocytes (Figure 5E-F). (Separate .xlsx file).
